# BACT-URIE: A Novel Score Integrating Bacteriuria to Predict Infective Endocarditis in Patients With *Staphylococcus aureus* Bacteremia

**DOI:** 10.1093/ofid/ofag259

**Published:** 2026-05-04

**Authors:** G Péan de Ponfilly, A Beresteanu, G Chatellier, M Cachanado, M Shima, A Mizrahi, A Le Monnier, B Pilmis

**Affiliations:** Service de Microbiologie Clinique, Hôpitaux Saint-Joseph & Marie-Lannelongue, Paris, France; Institut Micalis UMR 1319, Université Paris-Saclay, INRAe, AgroParisTech, Orsay, France; Equipe mobile de Microbiologie Clinique, Hôpitaux Saint-Joseph & Marie-Lannelongue, Paris, France; Département de recherche clinique, Hôpitaux Saint-Joseph & Marie-Lannelongue, Paris, France; Département de recherche clinique, Hôpitaux Saint-Joseph & Marie-Lannelongue, Paris, France; Département de recherche clinique, Hôpitaux Saint-Joseph & Marie-Lannelongue, Paris, France; Service de Microbiologie Clinique, Hôpitaux Saint-Joseph & Marie-Lannelongue, Paris, France; Institut Micalis UMR 1319, Université Paris-Saclay, INRAe, AgroParisTech, Orsay, France; Service de Microbiologie Clinique, Hôpitaux Saint-Joseph & Marie-Lannelongue, Paris, France; Institut Micalis UMR 1319, Université Paris-Saclay, INRAe, AgroParisTech, Orsay, France; Institut Micalis UMR 1319, Université Paris-Saclay, INRAe, AgroParisTech, Orsay, France; Equipe mobile de Microbiologie Clinique, Hôpitaux Saint-Joseph & Marie-Lannelongue, Paris, France

**Keywords:** bacteriuria, clinical prediction score, infective endocarditis, risk stratification, *Staphylococcus aureus* bacteremia

## Abstract

**Background:**

Infective endocarditis (IE) is a serious complication of *Staphylococcus aureus* bacteremia (SAB). Systematic echocardiographic evaluation, especially when including transesophageal echocardiography, is resource-intensive. Existing risk scores do not include *S. aureus* bacteriuria, a potential marker of hematogenous spread. We assessed whether bacteriuria improves IE risk stratification.

**Methods:**

We retrospectively analyzed 2 temporally distinct cohorts of adult SAB patients in a French tertiary hospital: a derivation cohort (2012–2019, n = 233) and a validation cohort (2020–2023, n = 156). Patients with proven urinary tract infection were excluded. Logistic regression identified predictors of IE, which were converted into weighted points to develop 2 scores: day 1 (baseline variables) and day 4 (including persistent bacteremia ≥72 hours). Performance was compared with VIRSTA and PREDICT.

**Results:**

Infective endocarditis occurred in 14.6% of the derivation cohort and 14.7% of the validation cohort. Independent predictors were intracardiac devices, community acquisition, bacteriuria, septic emboli, and persistent bacteremia. AUROCs for the day 1 and day 4 scores were 0.78 and 0.81 in derivation, and 0.91 and 0.93 in validation. At thresholds of >2 and >3, sensitivities were 67.65% and 70.59%, with negative predictive values of 93.29% and 93.83%, respectively. Compared with VIRSTA and PREDICT, BACT-URIE showed higher sensitivity in this cohort.

**Conclusions:**

BACT-URIE is an exploratory score incorporating *S. aureus* bacteriuria to predict IE in SAB. Prospective multicenter validation is required before clinical implementation.

## Teaser

The BACT-URIE score incorporates *S. aureus* bacteriuria to predict infective endocarditis in SAB patients. It offers strong negative predictive value and helps guide appropriate use of echocardiography.

*Staphylococcus aureus* is one of the leading causes of bloodstream infections in high-income countries, and is associated with substantial morbidity and mortality due to its propensity for metastatic infection, particularly infective endocarditis (IE) [1–3]. Infective endocarditis occurs in up to 20% of patients with *S. aureus* bacteremia (SAB) and substantially worsens prognosis [4]. International guidelines recommend systematic echocardiographic evaluation in all SAB cases, ideally including transesophageal echocardiography (TEE), to identify IE early and guide treatment [5–7]. However, this approach is resource-intensive, may be challenging to implement systematically in routine practice, and may result in unnecessary testing in low-risk patients. To optimize diagnostic strategies, several risk scores have been developed, such as VIRSTA and PREDICT, incorporating clinical and microbiological predictors to prioritize echocardiography [8–10]. The VIRSTA score combines variables such as septic emboli, meningitis, permanent intracardiac device or previous IE, preexisting native valve disease, intravenous drug use, community or non-nosocomial healthcare-associated acquisition, severe sepsis or shock or C-reactive protein > 190 mg/L [9]. The PREDICT score includes intracardiac device, onset of SAB and persistent bacteremia [11].

Despite their utility, these scores do not include *S. aureus* bacteriuria—an increasingly recognized phenomenon that may reflect hematogenous dissemination rather than primary urinary infection [12–15]. Up to one-third of SAB patients may present with concomitant bacteriuria, whose clinical interpretation has historically varied according to context [13]. Recent evidence suggests that its presence, especially in the absence of urinary symptoms, may reflect hematogenous seeding or a greater burden of disseminated infection [14, 15]. Its predictive value has not been formally integrated into existing decision tools. We hypothesized that *S. aureus* bacteriuria is an independent predictor of IE in SAB. We therefore aimed to develop and validate a novel scoring system (BACT-URIE) for early IE risk stratification, incorporating bacteriuria alongside clinical variables and persistent bacteremia. Our goal was to improve the precision of echocardiography indications while maintaining high diagnostic safety.

## METHODS

### Study Design and Setting

We conducted a 2-phase retrospective prognostic cohort study at the Hôpitaux Saint-Joseph & Marie-Lannelongue (Paris, France). The derivation cohort included adult patients hospitalized between January 2012 and December 2019, and the validation cohort included patients admitted between January 2020 and March 2023 at the same institution.

### Participants

Eligible patients were ≥18 years with at least one positive blood culture for *Staphylococcus aureus* and a urine culture obtained within 24 hours of the index blood culture. Patients with confirmed *S. aureus* urinary tract infection (UTI) were excluded. Urinary tract infection was defined using predefined clinical criteria and supportive objective findings suggestive of a primary urinary source, including urinary symptoms, pyuria, imaging findings when available, and a positive urine culture for *S. aureus* without another more likely source of bacteremia. Classification of UTI versus hematogenous bacteriuria was adjudicated by infectious diseases specialists from the study team, based on review of the clinical, microbiological, and imaging data. However, in the absence of urinary symptoms, distinguishing asymptomatic bacteriuria from hematogenous urinary seeding remained partly inferential. Using this definition, 15 patients were excluded. A prespecified sensitivity analysis including all patients with *S. aureus* bacteriuria (irrespective of UTI) was also performed.

Persistent bacteremia was defined as positive follow-up blood cultures at 72 hours, a pragmatic threshold chosen for early risk stratification in line with prior SAB literature [16]. All patients underwent transthoracic echocardiography (TTE). In addition, TEE was performed in 71/233 patients (30.5%) in the derivation cohort and in 45/156 patients (28.8%) in the validation cohort. Definite IE was adjudicated using the modified Duke criteria by infectious disease specialists [17].

### Data Collection

Demographic, clinical, laboratory, and microbiological data were collected from electronic medical records. Candidate variables considered for model development included: age, sex, diabetes, hemodialysis, cirrhosis, immunosuppression, intracardiac device, prosthetic hip or knee, Charlson comorbidity score, methicillin susceptibility, methicillin-resistant *Staphylococcus aureus* colonization, septic shock/ICU admission, acquisition setting (community, healthcare-associated, nosocomial), *S. aureus* bacteriuria, septic emboli, persistent bacteremia, and time-to-positivity of blood cultures.

### Assessment of Septic Emboli

Thoraco-abdomino-pelvic CT scan was performed in the absence of an obvious clinical source of bacteremia or when clinical symptoms prompted investigation. Cerebral imaging (CT or MRI) and spinal MRI were performed when clinically indicated.

### Microbiological Analysis

Blood cultures were incubated for 5 days, with extensions up to 10 days in suspected IE [18], according to French and international guidelines [17]. Time-to-positivity was defined as the interval between incubation start and detection of bacterial growth. Urine cultures and antimicrobial susceptibility testing were performed according to CA-SFM/European Committee on Antimicrobial Susceptibility Testing standards.

### Definition of Acquisition Setting

SAB was classified as:

- Community-acquired: first positive blood culture <48 hours after admission, without recent healthcare exposure;
- Healthcare-associated: blood culture <48 hours after admission plus recent hospitalization, dialysis, or outpatient IV therapy within 90 days;
- Nosocomia**l**: onset ≥48 hours after admission.

### Statistical Methods

#### Model Derivation

Infective endocarditis predictors were selected from the derivation cohort based on existing literature, including VIRSTA, and following variables were studied: age, sex, Charlson score, Intracardiac devices, Onset of SAB (Healthcare, Community, Nosocomial), Septic embolism, *S. aureus* bacteriuria, methicillin-resistant *S. aureus,* Time to blood culture positivity and persistent bacteremia.

Variables with *P* < .20 were entered into a multivariable logistic regression with backward stepwise selection, retaining variables with *P* < .05. The resulting predictors were used to construct 2 scores: the day 1 score (baseline variables) and the day 4 score (adding persistent bacteremia).

#### Internal Validation

Internal validation was performed via 1000 bootstrap iterations with backward selection. Predictors retained in >50% of samples were included in the final models. Discrimination was assessed using Harrell's c-statistic.

#### Risk Scores Calculation

To create bedside-usable risk scores, logistic regression coefficients (β) were rescaled into integer values (β’) by dividing by the smallest coefficient (β_s_) (β’=β/β_s_), multiplying by a constant (c = 2), and rounding to the nearest integer. Patient scores were calculated by summing the corresponding weights. Score performance (sensitivity, specificity, PPV [positive predictive value], negative predictive value [NPV]) was evaluated at different thresholds.

#### Temporal Validation

Validation was performed on a cohort of patients followed up later in the same hospitals. Thresholds were defined using the best Youden index (day 1 ≤ 2; day 4 ≤ 3), that is the greatest value of (sensitivity + specificity-1), classifying patients as negative (T−) or positive (T+). Sensitivity, specificity, PPV, and NPV were calculated to assess predictive performance for these thresholds.

Discrimination (area under the receiver operating characteristic curve [AUC]) refers to the ability of a predictive model to distinguish between individuals who develop the outcome of interest and those who do not. It was assessed for both the day 1 and day 4 models in the derivation and validation cohorts using the area under the receiver operating characteristic curve (AUC), that is, the c-statistic. Calibration evaluates the agreement between predicted probabilities and observed outcomes. It was assessed only in the validation cohort for both the day 1 and day 4 models using calibration plots, where predicted risks were plotted against observed event rates and visually inspected. Additionally, 2 numerical measures were computed: (1) Calibration-in-the-large, corresponding to the calibration intercept, which reflects systematic over-or underestimation of risk (ideal value = 0). (2) Calibration slope, which measures the strength of the relationship between predicted and observed risks across the risk spectrum. A slope of 1 indicates perfect calibration, while a slope < 1 suggests overfitting (predicted risks are too extreme), and a slope > 1 indicates underfitting.

More details were also available in the Supplementary Materials.

### Ethics and Data Protection

In accordance with the French Public Health Law (CSP Article L1121-1) and the General Data Protection Regulation (GDPR), the study received approval from the local ethics committee (IRB No. 00012157). Patients were informed that their data would be pseudonymized, securely stored, and used for research purposes. The database was registered with the French Data Protection Authority (CNIL), ensuring compliance and protection of personal information.

## RESULTS

### Study Population

During the study period, 248 patients were screened for inclusion in the derivation cohort. Among them, 15 had a primary *S. aureus* urinary tract infection and were therefore excluded according to prespecified criteria, leaving 233 patients included in the derivation analysis. Among them, 34 patients (14.6%) fulfilled the modified Duke criteria for definite IE. The median age was 75 years (interquartile range [66–84]), and 57.5% were male. Intracardiac devices were present in 13.7% of patients. All patients underwent TTE, and TEE was additionally performed in 71 patients (30.5%). *S. aureus* bacteriuria was identified in 40 patients (17.2%), of whom 10 developed IE. Persistent bacteremia occurred in 28.8% of patients. Regarding acquisition setting, 27.0% were community-acquired, 30.9% healthcare-associated, and 42.1% nosocomial. Among IE cases, 41.2% were community-acquired, 20.6% were healthcare-associated, and 38.2% were nosocomial. When stratified by acquisition setting, IE prevalence was the highest among patients with community-acquired SAB. Infective endocarditis occurred in 32.4% of patients with an intracardiac device and in 29.4% of those with concomitant *S. aureus* bacteriuria. Baseline characteristics by IE status are summarized in Table 1.

**Table 1.** Distribution of Candidate Variables by Infective Endocarditis Status in the Derivation Cohort

Baseline Candidate Variables	Total	IE	No IE	Univariate OR (95%CI)	*P* Value
	N = 233	N = 34	N = 199		
Age (year), median (IQR)	75.0 (66.0–84.0)	77.5 (66.0–88.0)	74.0 (65.0–84.0)	…	.32
Male sex, n (%)	134 (57.5)	20 (58.8)	114 (57.3)	1.07 [.51–2.23]	.87
Charlson score, mean (SD)	6.0 (3.1)	6.12 (3.14)	6.0 (3.1)*	…	.92
Intracardiac devices, n (%)	32 (13.7)	11 (32.4)	21 (10.6)	4.05 [1.73–9.47]	.0019
Onset of SAB					
Healthcare	72 (30.9)	7 (20.6)	65 (32.7)	1.00	.11
Community	63 (27)	14 (41.2)	49 (24.6)	2.65 [1.00–7.07]	
Nosocomial	98 (42.1)	13 (38.2)	85 (42.7)	1.42 [.54–3.76]	
Septic embolism, n (%)	14 (6.0)	9 (26.5)	5 (2.5)	13.97 [4.34 –45.00]	<.0001
*S. aureus* bacteriuria, n (%)	40 (17.2)	10 (29.4)	30 (15.1)	2.35 [1.02–5.40]	.0405
Methicillin-resistant *S. aureus*	20 (8.6)	3 (8.8)	17 (8.5)	1.04 [.29–3.75]	>.99
Time to blood culture positivity (h), mean (SD)	15.7 (9.3)	15.8 (12.5)	15.7 (8.6)	…	.31
Persistent bacteremia (≥72 h), n (%)	67 (28.8)	17 (50.0)	50 (25.1)	2.98 [1.42–6.28]	.0031

IE, infective endocarditis; SAB, *Staphylococcus aureus* bacteremia.

^*^1 missing data.

In the validation cohort (n = 156, 2020–2023), 23 patients (14.7%) developed definite IE. The median age was 74 years (IQR 63–86), and 64.1% were male. All patients underwent TTE, and TEE was additionally performed in 45 patients (28.8%). *S. aureus* bacteriuria was present in 41 patients (26.3%), including 11 with IE. Baseline characteristics of both cohorts are detailed in

### Multivariate Analysis and Score Construction

In multivariable logistic regression of the derivation cohort, 5 variables were independently associated with definite IE: the presence of an intracardiac device (OR 5.04, 95% CI 1.85–13.67), community-acquired SAB (OR 3.09, 95% CI 1.03–10.36), *S. aureus* bacteriuria (OR 3.23, 95% CI 1.21–8.46), septic emboli (OR 15.67, 95% CI 4.42–62.44), and persistent bacteremia ≥72 hours (OR 2.62, 95% CI 1.10–6.22).

Two prediction scores were derived from these predictors: day 1 score (baseline variables only: intracardiac device, acquisition setting, bacteriuria, septic emboli), ranging from 0 to 12 points and day 4 score (adding persistent bacteremia ≥72 hours), ranging from 0 to 15 points. Regression coefficients were rescaled and converted into integer points to facilitate bedside calculation (Table 2).

**Table 2.** Factors Independently Predictive of Infective Endocarditis and Model Coefficients of the 2 Risk Models (Derivation Cohort)

Model/Variable	Multivariable Odds Ratio (95% CI)	β	β’	C × β’	Points for the Bedside Model
Day 1 model				*c* = *2*	
Intracardiac device (yes vs no)	5.04 (1.85–13.67)	1.62	1.38	2.76	3
Onset of SAB					
Healthcare	1.0 (reference)	*…*	…	*…*	0
Nosocomial	2.00 (0.69–6.52)	0.69	0.59	1.18	1
Community	3.09 (1.03–10.36)	1.13	0.96	1.92	2
*S. aureus* bacteriuria (yes vs no)	3.23 (1.21–8.46)	1.17	1.00	2.00	2
Septic embolism (yes vs no)	15.67 (4.42–62.44)	2.75	2.34	4.69	5
					Score ranging from 0 to 12
Day 4 model				c = 2	
Intracardiac device (yes vs no)	5.47 (1.92–15.67)	1.70	1.77	3.53	4
Onset of SAB					
Healthcare	1.0 (reference)	*…*	…	*…*	0
Community	3.04 (0.99–10.37)	1.11	1.15	2.31	2
Nosocomial	1.87 (0.63–6.28)	0.63	0.65	1.31	1
*S. aureus* bacteriuria (yes vs no)	3.23 (1.18–8.60)	1.17	1.22	2.43	2
Septic embolism (yes vs no)	13.09 (3.52–54.26)	2.57	2.67	5.34	5
Persistent bacteremia (≥72 h vs < 72 h)	2.62 (1.10–6.22)	0.96	1.00	2.00	2
					Score ranging from 0 to 15

### Discrimination and Calibration

The discrimination and calibration results for both day 1 and day 4 models in the derivation and validation cohorts are summarized in Table 3.

**Table 3.** Discrimination and Calibration for Day 1 and Day 4 Models in the Derivation and Validation Cohorts

	Day 1 Model		Day 4 Model	
	Derivation Cohort	Validation Cohort	Derivation Cohort	Validation Cohort
C-statistic	0.78	0.91 (0.75 to 0.97)	0.81	0.93 (0.79 to 0.98)
Calibration in large	—	−0.70 (−1.24 to −0.16)	—	−0.75 (−1.32, −0.18)
Calibration slope	—	1.28 (0.84 to 1.73)	—	1.30 (0.85 to 1.76)

Calibration plots are shown in Supplementary Figures 1 and 2.

Discrimination was good to excellent in both cohorts. Interestingly, discrimination was higher in the validation cohort than in the derivation cohort for both the day 1 model (AUC 0.91 vs 0.78) and the day 4 model (AUC 0.93 vs 0.81), suggesting differences in case-mix rather than model overfitting. Concerning calibration, both the day 1 and day 4 models showed negative intercepts (−0.70 and −0.75, respectively), indicating a tendency to overestimate predicted risks (ie, on average, predicted probabilities were higher than the observed risks). Calibration slopes were greater than 1 (1.28 for the day 1 model and 1.30 for the day 4 model), suggesting underfitting: the models tend to overestimate high risks (too high predicted risk for patients at high observed risk) and underestimate low risks (too low predicted risk for patients at low observed risk).

### Clinical Utility and Comparison With Existing Scores

Diagnostic performance at predefined thresholds is detailed in Tables 4 and 5. The diagnostic performance of both the day 1 and Day 4 scores was assessed across various threshold values. Using a threshold of greater than 2 for the Day 1 score, the model yielded a sensitivity of 67.65%, a specificity of 76.88%, a NPV of 93.29%, and a PPV of 33.33%. When the day 4 score threshold was set above 3, sensitivity was 70.59%, specificity was 76.38%, NPV reached 93.83%, and PPV was 33.33%. Higher thresholds increased specificity and PPV but resulted in reduced sensitivity.

**Table 4.** **Performance of the 2 Scores for Identifying Infective Endocarditis in Patients With Staphylococcus** a**ureus Bacteremia, at Different Thresholds (Derivation Cohort)**

Score	Sensitivity (%)	Specificity (%)	False-Positive Rate (%)	False-Negative Rate (%)	Positive Predictive Value (%)	Negative Predictive Value (%)
Day 1 score						
0	91.18	22.61	66.09	1.29	16.76	93.75
1	85.29	54.77	38.63	2.15	24.37	95.61
2	67.65	76.88	19.74	4.72	33.33	93.29
3	47.02	86.93	11.16	7.73	38.10	90.58
4	38.24	96.48	3.00	9.01	65.00	90.14
5	26.47	97.99	1.72	10.73	69.23	88.64
7	11.76	99.5	0.43	12.88	80.00	86.84
8	11.76	100.00	0.00	12.88	100.00	86.90
9	5.88	100.00	0.00	13.73	100.00	86.15
10	0.00	100.00	0.00	14.59	NaN	85.41
Day 4 score						
0	94.12	16.58	71.24	0.86	16.16	94.29
1	91.18	39.70	51.50	1.29	20.53	96.34
2	82.35	62.81	31.76	2.58	27.45	95.42
3	70.59	76.38	20.17	4.29	33.80	93.83
4	55.88	88.94	9.44	6.44	46.34	92.19
5	41.18	94.97	4.29	8.58	58.33	90.43
6	35.29	97.49	2.15	9.44	70.59	89.81
7	23.53	98.49	1.29	11.16	72.73	88.29
8	14.71	98.99	0.86	12.45	71.43	87.17
9	8.82	98.99	0.86	13.30	60.00	86.40
10	8.82	99.50	0.43	13.30	75.00	86.46
11	9.82	100.00	0.00	13.30	100.00	86.52
13	0.00	100.00	0.00	14.59	NaN	85.41

**Table 5.** Diagnostic Performance of Day 1 and Day 4 Scores in Derivation vs Validation Cohorts Using Optimal Cutoffs

	Score Day 1 (>2)		Score Day 4 (>3)	
	Derivation	Validation	Derivation	Validation
Sensitivity	67.65%	91.30%	70.59%	91.30%
Specificity	76.88%	73.68%	76.38%	76.69%
Positive predictive value	33.33%	37.50%	33.80%	40.38%
Negative predictive value	93.29%	98.00%	93.83%	94.23%

#### Comparison With Existing Scores

**Figure 1.**
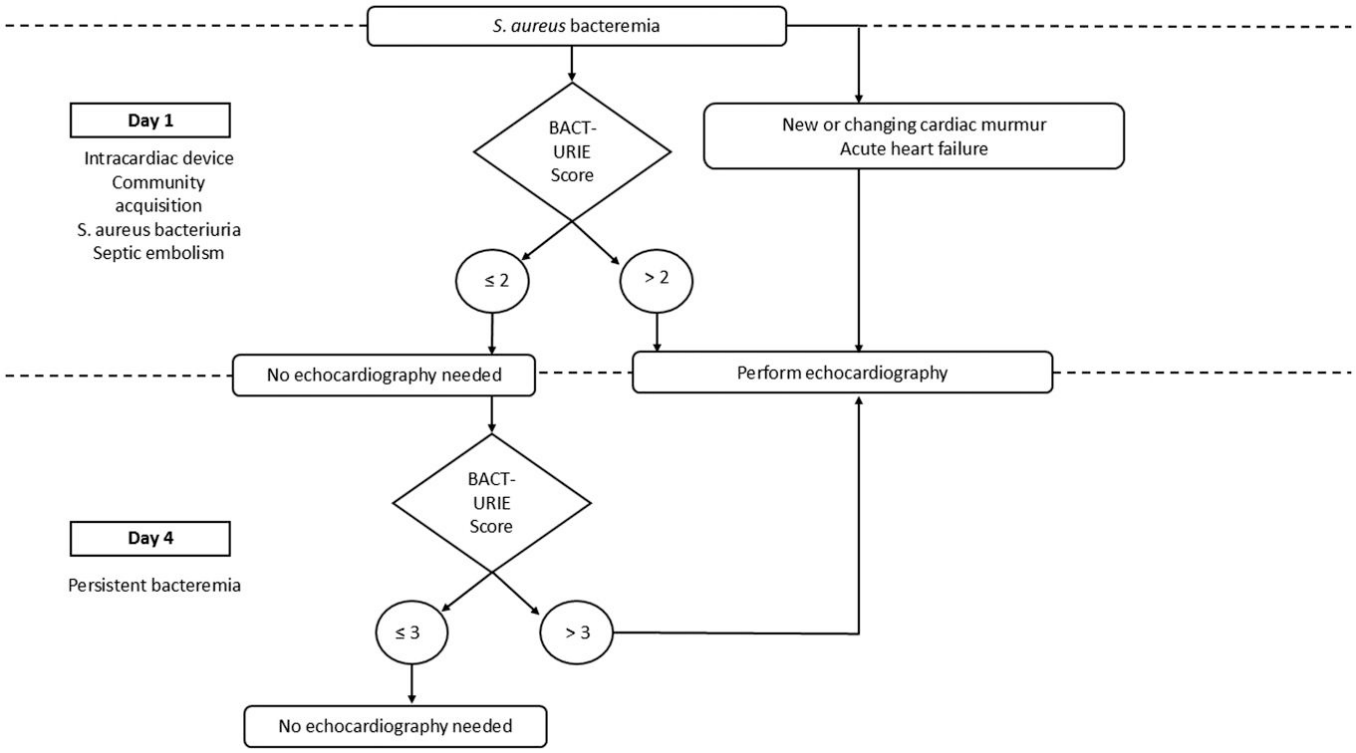
BACT-URIE–Based Decision Algorithm for Early Risk Stratification of Infective Endocarditis in SAB.

In the validation cohort, BACT-URIE, VIRSTA, and PREDICT showed different performance characteristics at their prespecified thresholds. VIRSTA and PREDICT yielded lower sensitivities and negative predictive values in our cohort than in several previously published validations. These differences should be interpreted cautiously, as they may reflect differences in case-mix, IE prevalence, referral patterns, and intensity of diagnostic workup. At their respective thresholds, the PREDICT day 1 score had a sensitivity of 41.2% and an NPV of 90.1%, while the PREDICT day 5 score achieved a sensitivity of 76.5% and an NPV of 92.2%. The VIRSTA score yielded a sensitivity of 41.2% and an NPV of 89.0%. Comparative data are presented in Table 6. BACT-URIE showed higher sensitivity at the selected thresholds in our cohort; however, these results should not be interpreted as demonstrating uniform superiority over existing tools. Based on this 2-step performance, we propose a clinical algorithm incorporating the BACT-URIE score at day 1 and day 4 to guide the use of echocardiography in patients with *S. aureus* bacteremia (Figure 1). Clinical judgment and a high degree of clinical suspicion should supersede any score-based recommendation, particularly in the presence of features such as a new cardiac murmur, acute heart failure, or other findings suggestive of IE.

**Table 6.** Performance of Various Prediction Score for Infective Endocarditis in the Validation Cohort

Score	Sensitivity	Specificity	Negative predictive value
PREDICT Day 1 (≥4)	41.2%	92.4%	90.1%
PREDICT Day 5 (≥2)	76.5%	47.9%	92.2%
VIRSTA (≥3)	41.2%	81.8%	89.0%
BACT-URIE Day 1 (>2)	91.3%	73.6%	98%
BACT-URIE Day 4 (>3)	91.3%	76.7%	94.2%

## DISCUSSION

Infective endocarditis is a frequent and severe complication of SAB, with a reported prevalence of 10%–20% in prospective cohorts [19, 20]. Early recognition remains challenging, as clinical presentation is often nonspecific and nearly half of IE cases occur in patients without classical risk factors [12]. Echocardiography, especially TEE, is critical for diagnosis but cannot be systematically performed in all SAB patients due to logistical and resource constraints. The main clinical challenge is therefore to identify, as early as possible, which patients truly require urgent cardiac imaging.

In this context, we developed and temporally validated the BACT-URIE score, a novel clinical tool to stratify the risk of IE in patients with *Staphylococcus aureus* bacteremia. Uniquely, our model incorporates *S. aureus* bacteriuria, a frequent but under-recognized microbiological finding. Up to one-third of SAB patients may present with concomitant bacteriuria, whose clinical interpretation has historically varied according to context [13, 14]. Our findings suggest that, in the absence of urinary symptoms or recent urological procedures, *S. aureus* bacteriuria may reflect hematogenous seeding or a greater burden of disseminated infection. In both the derivation and validation cohorts, bacteriuria was independently associated with IE.

The BACT-URIE score was designed as a 2-step strategy, with an early day 1 model based on baseline variables and a day 4 model incorporating persistent bacteremia. The day 1 score may provide an early pragmatic approach to risk stratification, whereas the day 4 score should be interpreted with particular caution, as a sensitivity around 70% is insufficient for a stand-alone rule-out strategy in routine practice. Accordingly, the score should not be used in isolation to defer echocardiography when clinical suspicion remains high. Rather than replacing clinical assessment, both versions should be viewed as exploratory tools that may help structure the evaluation of IE risk in SAB. An unexpected finding was the higher discriminative performance observed in the validation cohort compared with the derivation cohort. This difference should be interpreted cautiously and not overinterpreted, as it may reflect random variation related to the limited sample size and number of IE events, as well as possible differences in cohort composition. The validation cohort included a higher proportion of patients with septic emboli and *S. aureus* bacteriuria—2 strong predictors of IE—which may have contributed to this observation, but this remains speculative. Although calibration analyses did not suggest major overfitting, the temporal single-center nature of the validation limits firm conclusions regarding model transportability. Compared with existing prediction tools, BACT-URIE showed different performance characteristics in our cohort, but these differences should not be interpreted as evidence of uniform superiority. They may instead reflect cohort-specific factors, including case-mix, referral patterns, and intensity of diagnostic workup. The day 1 score has the advantage of being applicable early in the course of SAB, but its clinical usefulness remains to be confirmed in independent prospective cohorts.

Beyond overall performance, BACT-URIE identified a subgroup of patients with healthcare-associated SAB who had a low observed risk of IE. In our cohorts, 34/71 patients (48%) with healthcare-associated SAB had none of the 4 predictive variables, and only one patient in this subgroup developed IE. These findings suggest that deferring echocardiography may be reasonable in carefully selected patients with HCA-SAB; however, such decisions require confirmation in independent cohorts and must remain guided by overall clinical judgment.

Our results must also be interpreted in light of previous validations of existing scores. In their original derivation studies, both PREDICT and VIRSTA demonstrated very high sensitivities and NPVs, with PREDICT day 5 and VIRSTA low-risk groups yielding NPVs above 98% [8, 9, 21]. The distribution of IE risk according to acquisition setting in our cohort also differed from that reported for other tools such as VIRSTA and PREDICT, with an observed gradient of community-acquired > nosocomial > healthcare-associated SAB. This finding should be interpreted cautiously, as it may reflect cohort-specific characteristics rather than a generalizable pattern.

However, subsequent real-world validations have shown more modest performances. In a prospective Asian cohort of 634 SAB patients, Ngiam et al. reported that PREDICT day 5 ≥ 2 achieved only 58% sensitivity and VIRSTA ≥3 67% sensitivity, with NPVs around 96%–97% despite a relatively low IE prevalence of 5.7%. In our higher-prevalence cohort (∼15%), both PREDICT and VIRSTA performed even less well, with NPVs around 89%–92% and sensitivities as low as 41% at published cutoffs. By contrast, BACT-URIE showed higher sensitivities and NPVs than VIRSTA and PREDICT at the selected thresholds in our cohorts. These differences may reflect cohort-specific characteristics and require confirmation in independent external cohorts.

### Strengths and Limitations

Our study has several strengths. It is the first to formally evaluate *S. aureus* bacteriuria as a predictor of IE within a structured risk model. The 2-phase design, with derivation and temporal validation in 2 temporally distinct cohorts from the same institution, strengthens the internal consistency of our findings, although it does not establish independent external validity. The model was derived using rigorous methodology, including bootstrap internal validation, and benchmarked against established scores. Furthermore, echocardiographic evaluation was performed in all patients, limiting verification bias—a frequent limitation in SAB cohorts [17, 22].

Several limitations must be acknowledged. First, this was a retrospective single-center study, and the validation cohort, although temporally distinct, originated from the same institution; therefore, our findings do not constitute independent external validation and generalizability remains uncertain. Moreover, the validation period partly overlapped with the COVID-19 era, which may have influenced healthcare delivery, diagnostic pathways, and patient case-mix. Second, the number of IE events was modest, which may have limited model stability, particularly for less frequent predictors. Third, because inclusion required a urine culture obtained on the same day as the index blood culture, the study population may represent a more extensively investigated or clinically more severe subset of SAB patients, thereby limiting extrapolation to unselected SAB populations. Fourth, although patients with primary urinary tract infection were excluded using predefined clinical and supportive objective criteria, distinguishing asymptomatic bacteriuria from hematogenous urinary seeding remained challenging in this retrospective design, and some misclassification may have occurred. Fifth, TEE was not performed systematically: 71/233 patients (30.5%) in the derivation cohort and 45/156 (28.8%) in the validation cohort underwent TEE. Because TEE is the most sensitive echocardiographic modality for IE diagnosis, some cases may have remained undetected despite echocardiographic evaluation, potentially affecting the apparent performance of all evaluated scores. In addition, advanced imaging was not performed systematically; thoraco-abdomino-pelvic CT and cerebral or spinal MRI were obtained only when clinically indicated, which may have led to under-ascertainment of embolic complications at baseline. Finally, our study did not assess the impact of BACT-URIE-guided decision-making on clinical outcomes, delayed IE diagnosis, echocardiography use, or mortality. Accordingly, the real-world clinical impact of the score remains unknown.

## CONCLUSION

The BACT-URIE score is an exploratory 2-step tool for early IE risk stratification in patients with SAB. By incorporating *S. aureus* bacteriuria, it suggests that microbiological findings may add useful information to existing clinical prediction approaches. Although the score identified a subgroup of patients with low observed IE risk, it should not be used as a stand-alone rule-out tool, and echocardiographic decisions must remain guided by overall clinical assessment. Independent multicenter validation and prospective evaluation of clinical impact are required before implementation.

## Supplementary Material

ofag259_Supplementary_Data

## Supplementary Data

Supplementary materials are available at *Open Forum Infectious Diseases* online. Consisting of data provided by the authors to benefit the reader, the posted materials are not copyedited and are the sole responsibility of the authors, so questions or comments should be addressed to the corresponding author.
