# Supplementary material for: BACT-URIE: A Novel Score Integrating Bacteriuria to Predict Infective Endocarditis in Patients With Staphylococcus aureus Bacteremia
Source: Open Forum Infect Dis. 2026 May 4;13(5):ofag259. doi: 10.1093/ofid/ofag259 (PMC13202213; doi:10.1093/ofid/ofag259)

**Supplementary Figures**

**Figure S1 : Day 1 model calibration plot**


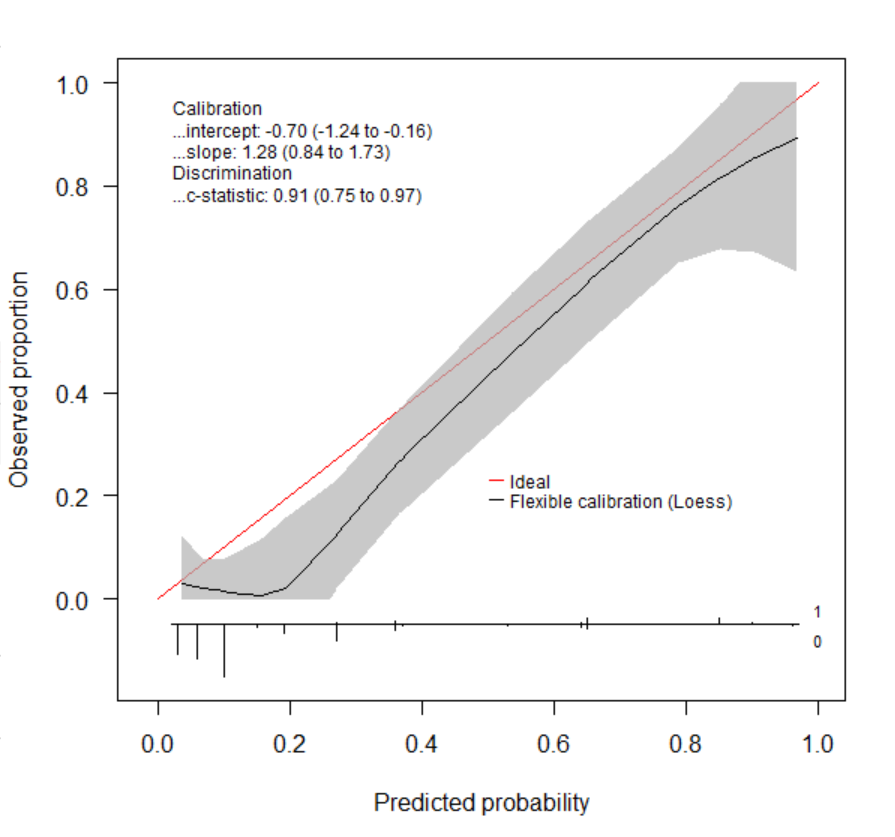


**Figure S2 : Day 4 model calibration plot**


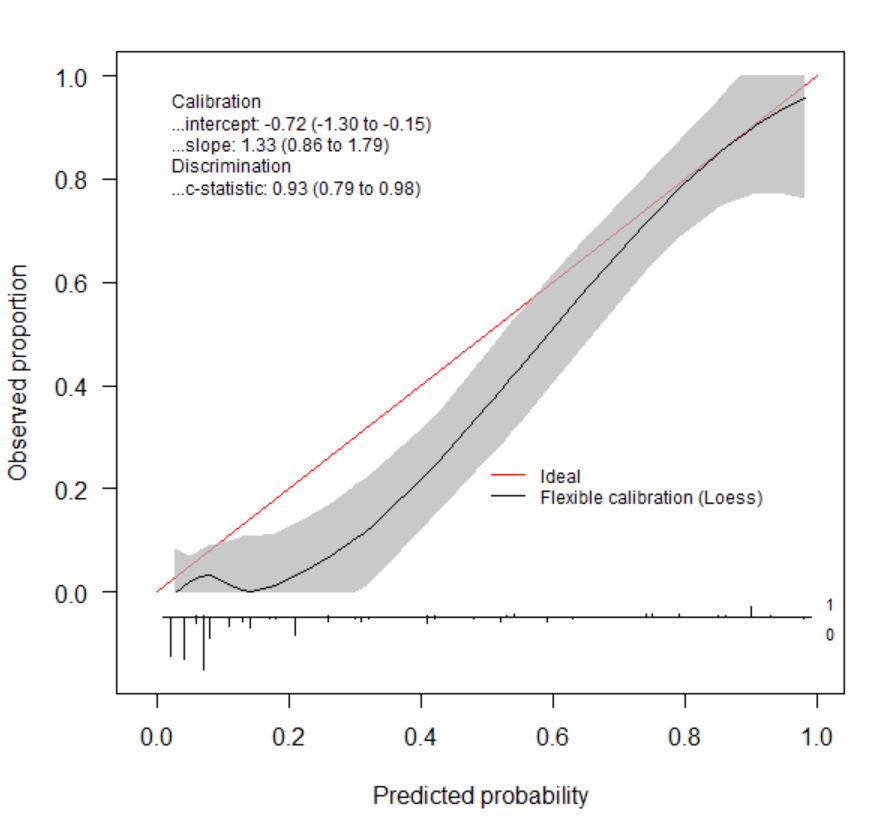

Supplement: ofag259_Supplementary_Data [file ofag259_supplementary_data.zip › Supplementary Figures.docx]
