# Supplementary material for: BACT-URIE: A Novel Score Integrating Bacteriuria to Predict Infective Endocarditis in Patients With Staphylococcus aureus Bacteremia
Source: Open Forum Infect Dis. 2026 May 4;13(5):ofag259. doi: 10.1093/ofid/ofag259 (PMC13202213; doi:10.1093/ofid/ofag259)

**Supplementary Tables**.

**Table S1** : **Description of Characteristics in Derivation and Validation Cohorts**

| Variable | Derivation cohort  (N= 233) | Validation cohort  (N = 156) | p-value |
| --- | --- | --- | --- |
| Age (yr), median (IQR) | 75 (66 – 84) | 74 (63 – 86) | 0.91 |
| Male sex, n (%) | 134 (57.5) | 100 (64.1) | 0.20 |
| Charlson score, mean (SD) | 6 (4 – 8) | 6 (5 – 7) | 0.78 |
| Intracardiac devices, n (%) | 32 (13.7) | 27 (17.3) | 0.38 |
| Onset of SAB |  |  |  |
| Community | 62 (26.6) | 50 (32.1) | 0.25 |
| Healthcare | 72 (30.9) | 50 (32.1) | 0.82 |
| Nosocomial | 99 (42.5) | 56 (35.9) | 0.2 |
| Septic embolism, n (%) | 14 (6) | 22 (14.1) | 0.01 |
| Echocardiography |  |  |  |
| TTE performed, n (%) | 233 (100) | 156 (100) |  |
| TEE performed, n(%) | 71 (30.5) | 45 (28.8) | 0.82 |
| *S. aureus* bacteriuria, n (%) | 40 (17.2) | 41 (26.3) | 0.04 |
| Methicillin-resistant *S. aureus* | 41 (17.6) | 21 (13.5) | 0.32 |
| Time to blood culture positivity (hours), mean (SD) | 13.9 (10.6 – 17.5) | 14.2 (11.3 – 18.1) | 0.74 |
| Persistent bacteremia (>= 72 hours), n (%) | 67 (28.8) | 37 (23.9) | 0.29 |

**Supplementary Figures**

**Figure S1 : Day 1 model calibration plot**


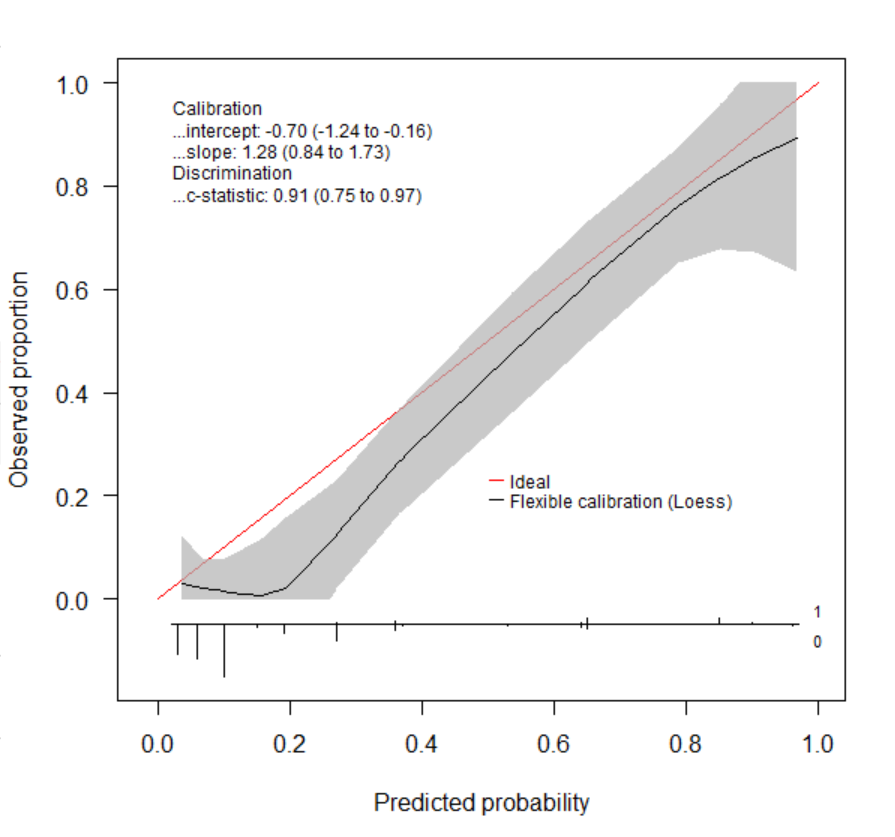


**Figure S2 : Day 4 model calibration plot**


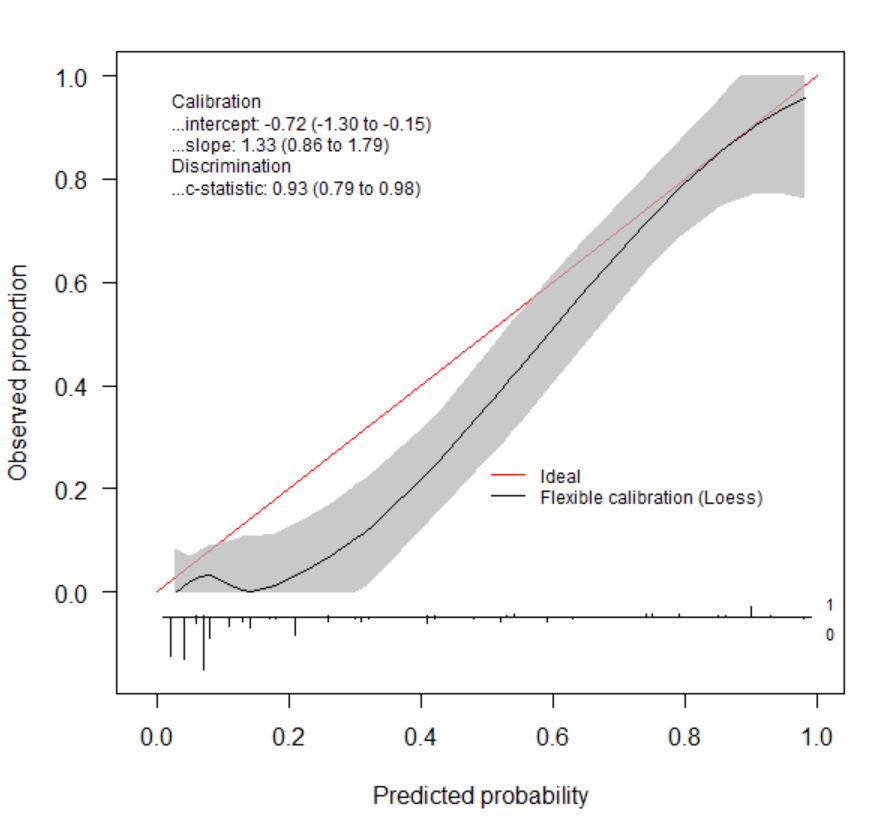

Supplement: ofag259_Supplementary_Data [file ofag259_supplementary_data.zip › Supplementary Tables.docx]
